# Effect of spaceflight on *Pseudomonas aeruginosa* final cell density is modulated by nutrient and oxygen availability

**DOI:** 10.1186/1471-2180-13-241

**Published:** 2013-11-06

**Authors:** Wooseong Kim, Farah K Tengra, Jasmine Shong, Nicholas Marchand, Hon Kit Chan, Zachary Young, Ravindra C Pangule, Macarena Parra, Jonathan S Dordick, Joel L Plawsky, Cynthia H Collins

**Affiliations:** 1 Department of Chemical & Biological Engineering, Rensselaer Polytechnic Institute, Troy, NY 12180, USA; 2 Center for Biotechnology and Interdisciplinary Studies (CBIS), Rensselaer Polytechnic Institute, Troy, NY 12180, USA; 3 Current address: Regeneron Pharmaceuticals Inc., Rensselaer, NY 12144, USA; 4 Lockheed Martin-Ames Research Center, Moffett Field, CA 94035, USA; 5 Department of Biology, Rensselaer Polytechnic Institute, Troy, NY 12180, USA

**Keywords:** Spaceflight, Microgravity, *Pseudomonas aeruginosa*, Flow cytometry, Motility

## Abstract

**Background:**

Abundant populations of bacteria have been observed on Mir and the International Space Station. While some experiments have shown that bacteria cultured during spaceflight exhibit a range of potentially troublesome characteristics, including increases in growth, antibiotic resistance and virulence, other studies have shown minimal differences when cells were cultured during spaceflight or on Earth. Although the final cell density of bacteria grown during spaceflight has been reported for several species, we are not yet able to predict how different microorganisms will respond to the microgravity environment. In order to build our understanding of how spaceflight affects bacterial final cell densities, additional studies are needed to determine whether the observed differences are due to varied methods, experimental conditions, or organism specific responses.

**Results:**

Here, we have explored how phosphate concentration, carbon source, oxygen availability, and motility affect the growth of *Pseudomonas aeruginosa* in modified artificial urine media during spaceflight. We observed that *P. aeruginosa* grown during spaceflight exhibited increased final cell density relative to normal gravity controls when low concentrations of phosphate in the media were combined with decreased oxygen availability. In contrast, when the availability of either phosphate or oxygen was increased, no difference in final cell density was observed between spaceflight and normal gravity. Because motility has been suggested to affect how microbes respond to microgravity, we compared the growth of wild-type *P. aeruginosa* to a *Δ*motABCD mutant deficient in swimming motility. However, the final cell densities observed with the motility mutant were consistent with those observed with wild type for all conditions tested.

**Conclusions:**

These results indicate that differences in bacterial final cell densities observed between spaceflight and normal gravity are due to an interplay between microgravity conditions and the availability of substrates essential for growth. Further, our results suggest that microbes grown under nutrient-limiting conditions are likely to reach higher cell densities under microgravity conditions than they would on Earth. Considering that the majority of bacteria inhabiting spacecrafts and space stations are likely to live under nutrient limitations, our findings highlight the need to explore the impact microgravity and other aspects of the spaceflight environment have on microbial growth and physiology.

## Background

Bacteria can sense changes in their surroundings and modulate their gene expression and physiology to adapt to a range of environmental conditions. A unique condition that bacteria encounter during spaceflight is significantly reduced gravity, known as microgravity [1,2]. Many studies have shown that the microgravity environment encountered during spaceflight can alter bacterial physiology, including increased growth rate, antibiotic resistance, and virulence [3]. The effects of microgravity on final cell density have been described for several bacterial species, including *Escherichia coli*[4-9], *Bacillus subtilis*[10,11], and *Salmonella enterica* serovar Typhimurium (hereafter referred to as *Salmonella*) [12,13]. A number of these studies have shown increased final cell density during spaceflight, while others have not observed significant differences between spaceflight and normal gravity conditions [14]. Interestingly, independent studies of the same species have also yielded differing results, where some examining *E. coli* during spaceflight have reported increased final cell density [5,8], and others have reported no differences relative to normal gravity controls [4,6]. Some studies have been repeated on multiple shuttle missions and yielded consistent results [13,15], suggesting that these differences are likely not due to issues with experimental design or reproducibility. As such, we hypothesize that these discrepancies may be attributed to differences in media and culture conditions, in addition to differences in how individual species respond to the microgravity environment.

Benoit and Klaus have suggested that differences in motility may be responsible for some of these inconsistent observations [14]. They hypothesize non-motile bacteria have an advantage under spaceflight conditions due to a lack of cell settling and the resulting increases in local nutrient availability in the well-mixed environment, while motile cells are able to move actively towards nutrients under both normal and microgravity conditions [14]. Indeed, a previous spaceflight experiment, conducted by Thévenet *et al.,* found that growth in microgravity increased the final cell density of a non-motile *E. coli* mutant lacking *motB* relative to normal gravity, while no differences were observed with the motile parent [9]. However, further studies are needed to examine whether motility plays a universal role in affecting microbial growth in microgravity.

Recent studies have also indicated that media conditions can modulate how microgravity affects bacterial physiology [15-17]. *Salmonella* cultured in Lennox broth (LB) during spaceflight exhibited increased virulence in a murine model of infection compared to controls where *Salmonella* was grown on Earth. However, the use of M9 media or the addition of the inorganic ion components of M9 to LB was observed to mitigate the enhanced virulence of *Salmonella* induced during spaceflight [15]. A set of follow-up experiments tested the individual components of M9 and showed that the addition of phosphate alone to LB could prevent the increase in acid tolerance observed when *Salmonella* is grown in low shear modeled microgravity (LSMMG) [15].

In addition to phosphate, oxygen availability may also influence how bacteria grow and respond to the spaceflight environment. For example, oxygen is known to affect many aspects of the growth and physiology of *Pseudomonas aeruginosa*, a facultative anaerobe, including growth, motility, and biofilm formation [18-20]. *P. aeruginosa* is an opportunistic human pathogen and was attributed to the spaceflight infection of an astronaut during Apollo 13 [21,22]. We recently examined biofilm formation by *P. aeruginosa* PA14, a virulent clinical isolate [23], and shown that differences in biofilm biomass observed between spaceflight and normal gravity can be minimized when oxygen availability is increased [24]. Modeling efforts have suggested that oxygen transfer rates may be decreased under LSMMG conditions compared to normal gravity conditions [16], and a number of genes involved in anaerobic metabolism were found to be up-regulated when *P. aeruginosa* PAO1, a less virulent laboratory reference strain [25,26], was grown during spaceflight [17]. These findings indicate that oxygen availability may influence bacterial responses to spaceflight, and highlight the interplay between changes in the environment due to the direct or indirect effects of microgravity, such as reduced oxygen transport, and changes in microbial physiology. However, previous studies have not examined changes in the response of planktonic cells to the spaceflight environment when oxygen availability is varied.

The methods used to investigate microbial growth during spaceflight may also be responsible for some of the differences. To date, the measurement of bacterial cell density has most often been conducted after samples were returned to Earth due to constraints imposed by the nature of such spaceflight experiments [27]. The two methods of storing planktonic samples of bacteria grown during spaceflight prior to analysis used most frequently are the addition of a fixative to the culture [6,9,28] and the storage of live samples at low temperatures [4,5]. In the present study, we assess the use of two methods for measuring microbial cell concentrations following growth during spaceflight: plate counting of live samples and flow cytometry (FCM) of fixed samples. Plate counting has the advantage of allowing the measurement of viable cell numbers. FCM is a fast and precise method to count cells [29]. Furthermore, this method is able to directly count hundreds of thousands of cells in a single sample, thereby facilitating the generation of statistically meaningful results.

We have investigated the effects of phosphate concentration, carbon source, oxygen availability and swimming motility on the final cell density of *P. aeruginosa* PA14 grown during spaceflight. Here, we demonstrate that the final cell density of *P. aeruginosa* is increased during spaceflight when culture conditions limit the availability of substrates required for growth. Specifically, growth during spaceflight led to an increase in final cell density when *P. aeruginosa* was cultured under low phosphate and low oxygen conditions. Increased availability of either phosphate or oxygen was observed to minimize the effects of spaceflight on the final cell density. These findings indicate that effects of gravity on final cell density can be modulated by the availability of growth-limiting substrates.

## Results

### Two assays of final cell density show inconsistent measurements of spaceflight and normal gravity cultures grown in specialized hardware

To examine how spaceflight affects final cell density, *P. aeruginosa* PA14 was grown aboard the Space Shuttle Atlantis during STS-135 in specialized hardware, known as a fluid processing apparatus (FPA). FPAs have been used extensively to culture microbes during spaceflight [8,13,15,17,24,30]. As shown (Additional file 1: Figure S1), FPAs are glass barrels with a bevel on the side. Rubber stoppers are added to form compartments and the bevel enables mixing of the fluids from the different compartments via a plunging action. The first compartment was loaded with modified artificial urine media (mAUM), which was used because it provides a physiologically relevant environment for the study of bacterial growth [31,32]. Understanding how microbes grow in urine aboard a spacecraft or space station may be important for minimizing the potential for both future infections and microbial-based failures of the essential water and waste purification systems. Further, because mAUM is a chemically defined medium, it can be easily manipulated to test effects of specific compounds on final cell density. The second compartment was loaded with cells used for inoculation stored in PBS. For fixed samples, a third compartment was filled with a 9% w/v paraformaldehyde solution in PBS.

**Figure 1:**
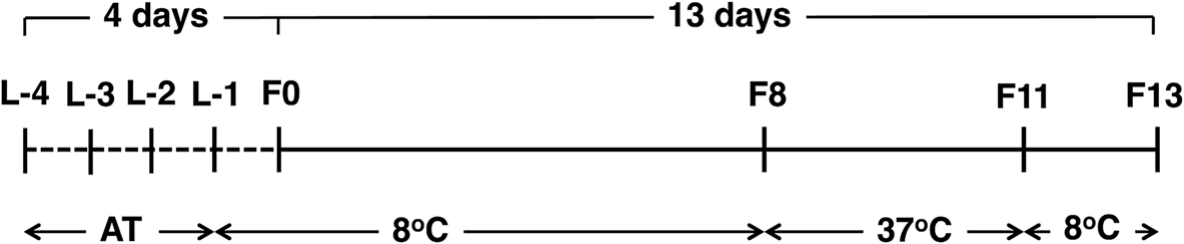
**Timeline of spaceflight experiments.** Four days before Shuttle launch (L-4): Media were loaded into FPAs. L-3: Inocula were loaded. L-2: After checking for contamination, 9% (w/v) paraformaldehyde fixative was loaded for FCM samples only. Samples were stored at ambient temperature (AT) from L-4 to L-1. L-1: After a final contamination check, samples were loaded into the CGBA and stored at 8°C. F0: Space Shuttle Atlantis launched. F8: Samples were activated by mixing the media with the inocula. The temperature in the CGBA was increased to 37°C. F11: Samples were terminated by the addition of fixative (FCM samples only) 72 h after activation. The temperature in the CGBA was reduced to 8°C. F13: Space Shuttle Atlantis landed. Ground controls were conducted at Kennedy Space Center in parallel with spaceflight samples.

Cultures were grown following the experiment timeline shown in Figure 1. Prior to inoculation, the FPAs were stored at 8°C. Following inoculation, cells were cultured at 37°C for 72 h. A subset of the cultures were fixed with paraformaldehyde immediately following the 72 h culture period and all cultures were subsequently incubated at 8°C for approximately 48 h. This 48 h period corresponds to the time required for the shuttle to return to Earth, and storage at 8°C during this period minimized additional cell growth of viable cultures and increased the stability of the fixed samples [6,33]. Plate counting was conducted immediately following shuttle landing to determine the number of viable cells in the live samples. The paraformaldehyde-fixed samples were stained with DAPI and cell numbers were determined by flow cytometry (FCM).

**Figure 2:**
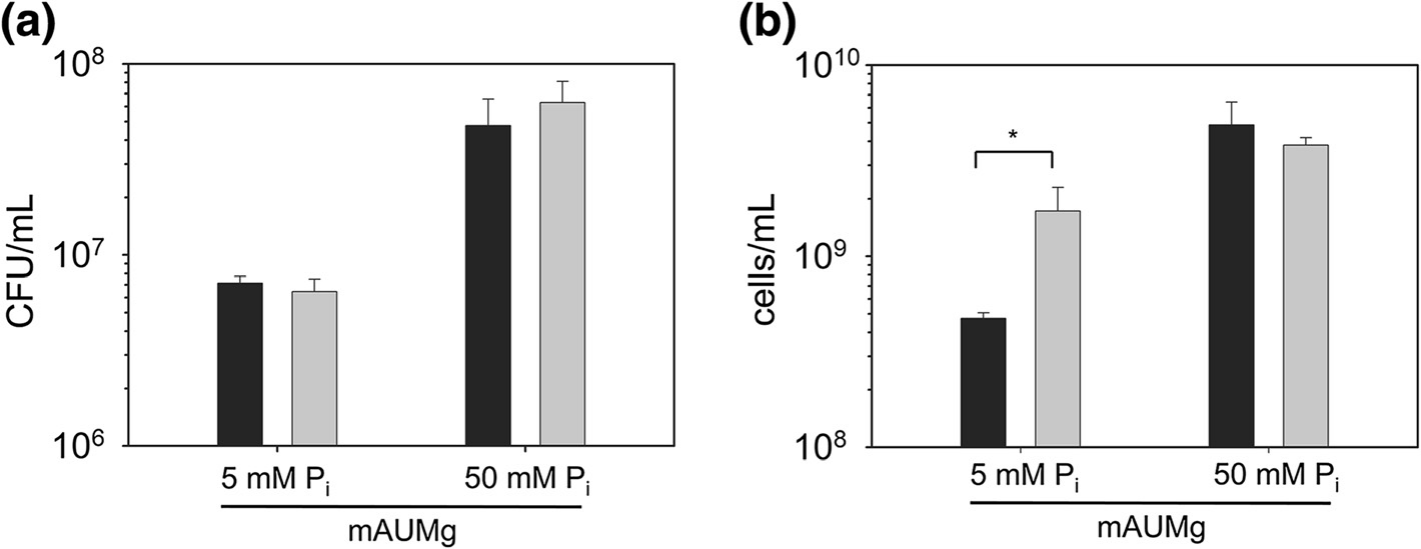
**Comparison of plate counting and FCM measurements of microbial growth during spaceflight.** Wild-type *P. aeruginosa* was cultured under normal gravity (black bars) and spaceflight (gray bars) conditions in mAUMg containing 5 mM or 50 mM phosphate. **(a)** The number of viable cells in unfixed samples was measured by plate counting. **(b)** The number of cells in fixed samples was measured by flow cytometry (FCM). Statistical differences between normal gravity and spaceflight for each experimental condition were analyzed by one-tailed t-test: *p < 0.05. Error bars indicate SD; N = 3.

While we anticipated data from plate counting and FCM measurements of final cell concentration would yield similar results and reinforce each other, our analysis showed very different results from the two methods under a number of conditions. This discrepancy between the two methods was most pronounced when cells were cultured in mAUM containing 2 mM glucose (mAUMg). In mAUMg, FCM data indicated that growth during spaceflight led to increased cell numbers compared to normal gravity, while plate counting data showed no difference in the final cell density between spaceflight and normal gravity (Figure 2). However, when the phosphate concentration was increased from 5 to 50 mM (mAUMg-high Pi), FCM and plate counting data exhibited similar trends (Figure 2).

**Figure 3:**
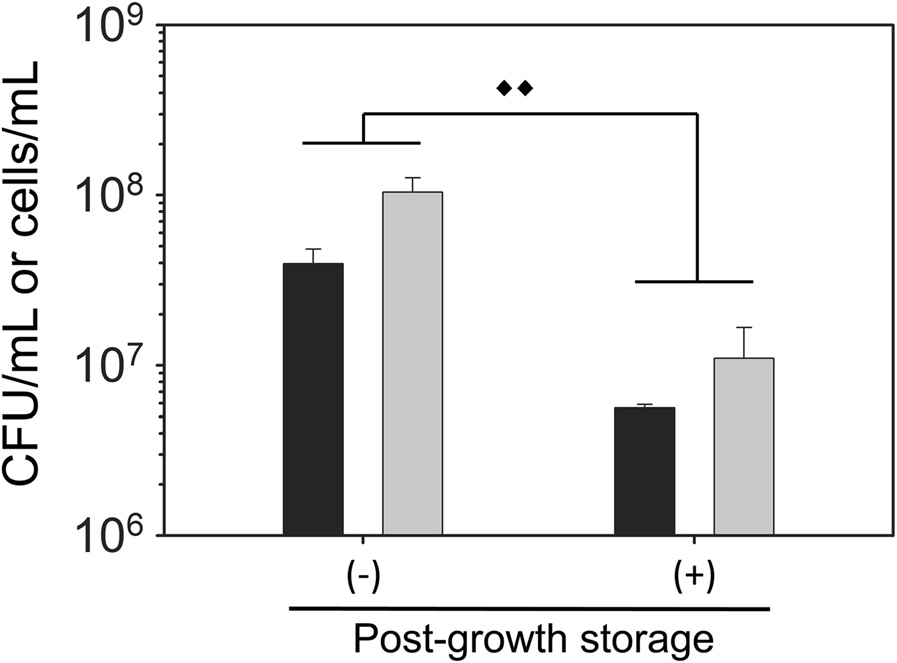
**Post-growth storage of samples at 8°C for 48 h decreases final cell density.** Wild-type *P. aeruginosa* was cultured in mAUMg containing 5 mM phosphate at 37°C for 72 h. Final cell density was measured immediately following growth (-) or following post-growth storage at 8°C for 48 h (+) by plate counting (black bars) or FCM (gray bars). For FCM samples, fixative was added immediately following growth (-) or after post-growth storage at 8°C for 48 h (+), and cells were stored at 4°C prior to staining and assessing cell numbers. Diamonds indicate results from two-way ANOVA: ♦♦*p <* 0.01. Error bars, SD; N = 3.

In order to resolve the discrepancies between plate counting data and FCM data, we conducted additional ground-based experiments where we focused on the effects of storing cells at 8°C following cell growth. Post-growth storage was targeted because bacterial storage at low temperatures is known to affect bacterial physiology and viability [34,35]. Although the FCM samples were also stored at 8°C for 48 h following cell growth, we note that the paraformaldehyde fixative was added prior to lowering the incubator temperature. To test the role of post-growth storage conditions on cell viability, we compared final cell concentrations obtained when cells were plated immediately following growth, or after post-growth storage at 48 h at 8°C. The same timeline and temperature profile from the spaceflight experiments (Figure 1) was followed. As shown in Figure 3, results from both plate counting and FCM showed that *P. aeruginosa* cultured in mAUMg exhibited a 7- to 9-fold decrease in cell numbers following storage at 8°C for 48 h (*p* < 0.01, two-way ANOVA). Our results indicate that measurements of cell numbers following storage at low temperatures post-growth may not accurately represent the amount of growth that occurred in the FPAs, and differences in final cell densities observed between spaceflight samples and ground controls may be confounded by differences in death rates. Because it is not currently feasible to plate count directly following the growth period during spaceflight, FCM measurements of cells fixed immediately following growth provide more reliable information with respect to how much biomass was formed during the growth period. Hereafter, we will describe only the results obtained by FCM of samples fixed immediately after the growth period.

### Phosphate availability modulates the effect of spaceflight on final cell density

**Figure 4:**
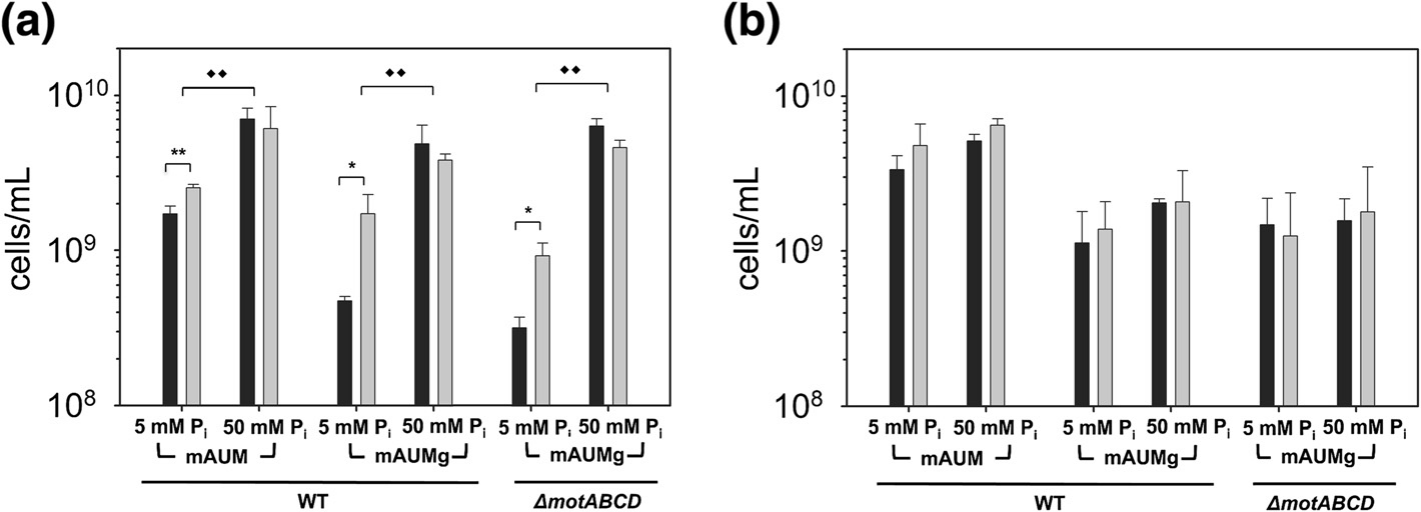
**Effect of spaceflight on final cell density of *P. aeruginosa*.** Wild-type *P. aeruginosa* and a motility mutant (*ΔmotABCD*) were cultured under normal gravity (black bars) and spaceflight conditions (gray bars) in FPAs with **(a)** solid inserts or **(b)** GE inserts. Cells were cultured in mAUM or mAUMg containing 5 mM or 50 mM phosphate. Final cell density was measured by FCM. Effect of phosphate on final cell density is significant in FPAs with solid inserts [two-way ANOVA, ♦♦*p* < 0.01]. Effect of gravity on final cell density is significant in 5 mM phosphate conditions in FPAs with solid inserts [two-way ANOVA, *p* < 0.01]. Statistical differences between spaceflight and normal gravity for each experimental condition was analyzed by one-tailed t-test: **p < 0.01; *p < 0.05. Error bars indicate SD; N = 3.

*P. aeruginosa* grown in mAUM during spaceflight exhibited a modest, but significant, 1.5-fold increase in final cell density compared to normal gravity controls (*p <* 0.01*,* one-tailed t-test) (Figure 4(a)). We also measured final cell densities in mAUM where the concentration of phosphate was increased from 5 mM, which is the concentration found in normal human urine [32], to 50 mM (mAUM-high Pi). In the case of mAUM-high Pi, no difference in the final cell density was observed between spaceflight and normal gravity (Figure 4(a)).

In addition to phosphate, we also examined the effect of carbon source on the response of *P. aeruginosa* to the spaceflight environment. Changing carbon source is known to affect *P. aeruginosa* metabolism and growth rate [36-38]. To assess the effect of carbon source on *P. aeruginosa* growth during spaceflight, we substituted the 2 mM citrate found in mAUM with 2 mM glucose (mAUMg). As shown in Figure 4(a), *P. aeruginosa* grown in mAUMg during spaceflight showed a 4-fold increase in final cell density compared to normal gravity controls (*p <* 0.05, one-tailed t-test). Similar to mAUM-high Pi, no difference between final cell densities in spaceflight and normal gravity cultures was observed when the phosphate concentration was increased to 50 mM (mAUMg-high Pi) (Figure 4(a)). These results show that while substituting citrate with glucose does not affect the final cell density of *P. aeruginosa* grown during spaceflight, altering phosphate concentration does affect final cell density (*p <* 0.01, two-way ANOVA), and that there is an interaction between the microgravity environment and phosphate concentration, as factors affecting final cell density, that is statistically significant (*p <* 0.05, two-way ANOVA). This finding indicates that increasing phosphate availability can minimize the effect of spaceflight on final cell density of *P. aeruginosa*.

### Oxygen availability modulates the effect of spaceflight on final cell density

We hypothesized that the effects of spaceflight on final cell density might differ between anaerobic and aerobic conditions. To test this hypothesis, we substituted the solid inserts used in the experiments described above with gas exchange (GE) inserts that allow the movement of gases via a gas-permeable membrane. GE inserts have been used previously to increase the oxygen availability and promote aerobic growth in FPAs [13,15,17,39]. Unlike cultures grown with solid inserts (Figure 4(a)), *P. aeruginosa* cultured in mAUM with GE inserts showed no difference in final cell density between normal gravity and spaceflight regardless of phosphate concentration (Figure 4(b)). Similar results were obtained for samples with GE inserts cultured in mAUMg and either 5 or 50 mM phosphate (Figure 4(b)). These results indicate that, similar to phosphate, increased oxygen availability can reduce the effects of spaceflight on final cell density.

### Role of swimming motility on spaceflight final cell density

We also examined the effects of spaceflight on a motility mutant, *ΔmotABCD,* which lacks the motor proteins required for flagella-mediated swimming [40]. In mAUMg, *ΔmotABCD* cultured during spaceflight showed a 3-fold increase in final cell density compared to its normal gravity counterpart (*p* < 0.05, one-tailed t-test), while in mAUMg-high Pi there was no difference in final cell density (Figure 4(a)). We also cultured *ΔmotABCD* with GE inserts and observed no difference in the final cell density between spaceflight and normal gravity controls, regardless of phosphate availability (Figure 4(b)). Overall, we did not observe any significant differences between the final cell density of wild-type *P. aeruginosa* and the non-motile mutant under any of our experimental conditions (*p >* 0.05, three-way ANOVA; factors: gravity, phosphate, motility).

### Microscopy of planktonic cells does not show aggregation of *P. aeruginosa* PA14

**Figure 5:**
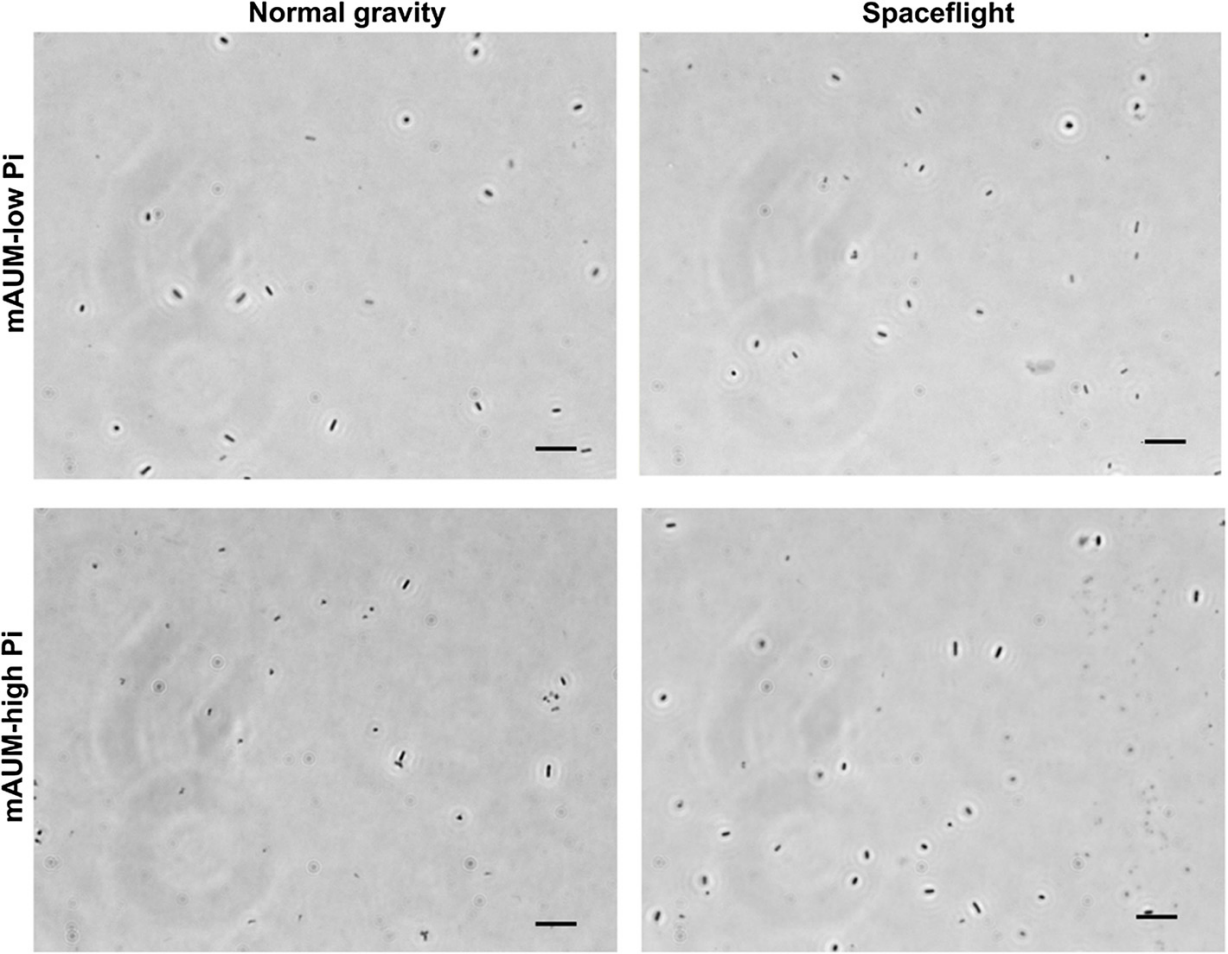
**Absence of aggregation by planktonic cells grown in FPAs.** Representative images obtained by DIC microscopy of paraformaldehyde-fixed *P. aeruginosa* cells grown in mAUM containing 5 mM phosphate (top) or 50 mM phosphate (bottom) during normal gravity and spaceflight. Scale bar is 10 μm.

Increases in aggregation and clumping of planktonic cells have been observed for *Salmonella* grown during spaceflight [13] and *P. aeruginosa* PAO1 grown in LSMMG [41]. To assess whether spaceflight conditions induced cellular aggregation in our spaceflight experiments, we observed the fixed *P. aeruginosa* PA14 planktonic cells by differential interference contrast (DIC) microscopy. Interestingly, no clumps or aggregates with more than ~3 cells were observed following growth in mAUM with 5 or 50 mM phosphate during spaceflight or on Earth (Figure 5), or under any of the other media conditions tested (data not shown).

## Discussion

We have shown that, within the constraints of our spaceflight experiments, FCM of fixed cells provides more reliable measurements of final cell numbers of planktonic cells grown in FPAs during spaceflight and on Earth. Although fixing the cells prevents one from distinguishing between live and dead populations, the effects of post-growth storage at 8°C on cell viability indicate that the viability of cells during post-growth storage should be assessed on Earth prior to spaceflight studies, and suggest that the use of viable cell measurements should be avoided when possible. Further, a recent study has shown that changes in growth rate that occur when *Micrococcus luteus* is cultured in simulated microgravity conditions can be lost within 7 h of exposure to normal gravity [42]. Changes in resistance to oxidative stress were similarly lost within 2 h after *Staphylococcus aureus* cells were removed from LSMMG conditions [43]. Of course, for certain spaceflight studies, such as those assessing changes in virulence or antibiotic resistance, the use of viable cells is essential. Interestingly, the observation that *Salmonella* virulence is increased following growth during spaceflight [13], which has been confirmed by both additional spaceflight and simulated microgravity experiments [15], indicates that at least some of the physiological changes that occur during spaceflight are maintained when the samples are returned to Earth and assayed or used immediately. In the *Salmonella* virulence studies, Wilson and co-workers stored cells in fresh media at ambient temperature following growth during spaceflight [13,15]. In our experiments we did not use this approach in order to avoid increases in cell number due to the additional media. Together these results highlight the careful, nuanced approach that must be taken in order to conduct spaceflight experiments that provide results that truly reflect how the cells grow and adapt to the spaceflight environment.

We have systematically studied the effects of spaceflight on the final cell density of *P. aeruginosa,* assessing factors known to affect bacterial growth such as phosphate and oxygen availability, carbon source and motility. We have demonstrated that differences between final cell density of *P. aeruginosa* cultured during spaceflight and on Earth are dependent on nutrient and oxygen availability. Specifically, spaceflight increased the final cell density of *P. aeruginosa* when it was grown with low phosphate and low oxygen availability. When either phosphate or oxygen availability was increased, no difference between normal gravity and spaceflight was observed.

Similar to previous observations that increasing phosphate concentration or supplementing media with the inorganic ion components of M9 media, which includes phosphate, can minimize the effects of spaceflight or LSMMG on virulence and acid tolerance of *Salmonella*[15], we observed that increasing the phosphate concentration from 5 to 50 mM in the artificial urine media minimized the differences between spaceflight and normal gravity final cell densities of *P. aeruginosa*. Berry *et al.*, have also shown that phosphate uptake by *Saccharomyces cerevisiae* increased when cultured during spaceflight [44]*.* Taken together, these findings may indicate that phosphate plays a key role in microbial responses to spaceflight conditions. However, additional studies are needed to determine whether phosphate is acting simply as a growth-limiting nutrient, or if the effect is more specific. While cells grown with 5 mM phosphate are not likely to be starved for this essential nutrient, increasing phosphate concentration has been shown to increase the growth rate of *P. aeruginosa*[45]. We also observed that using GE inserts minimized differences between final cell concentrations following growth under normal and microgravity. Similarly, Wilson *et al.*, did not observe any difference in final cell concentration between spaceflight and normal gravity when *Salmonella* was cultured in FPAs with GE inserts, as measured by FCM [13,15,17]. While we observed that the use of GE inserts was advantageous for bacteria grown in normal gravity, no additional increases were observed for spaceflight cultures when the solid inserts were replaced with the GE inserts. This may be attributed to a lower oxygen transfer rate in microgravity, as described previously for LSMMG [16]. Indeed, studies of *P. aeruginosa* PAO1 gene expression have indicated that growth in FPAs is likely microaerophilic or anaerobic even with the GE inserts and that differences in oxygen gradients within the FPAs likely exist between normal gravity and spaceflight conditions [17]. We have recently observed that the use of GE inserts also minimized differences in *P. aeruginosa* PA14 biofilm growth between normal and spaceflight conditions [24]. In that case, we did see increases in biofilm biomass in spaceflight samples with GE inserts. However, the proximity of the biofilm substrate to the GE inserts ensured that any additional oxygen was delivered directly to the growing biofilm cells. Overall, these findings highlight the role that environmental conditions and nutrient availability have on modulating how microbes respond to the spaceflight environment.

While motile bacteria have been predicted to be less affected by the indirect effects of gravity on Earth, such as cell settling, due to their ability to self-propel [14], we did not observe any difference between the final cell densities of motile and non-motile *P. aeruginosa*. This is likely due to the fact that even motile *P. aeruginosa* do not swim constantly*.* Indeed, bacterial motility has been shown to vary during growth [46], where the proportion of cells actively swimming decrease as bacteria approach stationary phase [47]. As such, the difference between our results and earlier experiments with *E. coli*[9] may be attributed to differences in the regulation of swimming motility between the two organisms under the conditions tested. Together, this suggests that while a constantly swimming population of cells may mitigate the role of gravity, the regulation of motility can lead even cells that are capable of swimming motility to be affected by gravity and to exploit the advantages of the microgravity environment.

Unlike *P. aeruginosa* PAO1 cultured under LSMMG, *P. aeruginosa* PA14 cultured during spaceflight did not form cellular aggregates in planktonic cultures. This observation is likely due to differences in the extracellular polymeric substances (EPS) that are responsible for cellular aggregation [48]. Production of EPS in *P. aeruginosa* is known to be modulated by various environmental factors, including shear stress [49]. The difference between the LSMMG results and our spaceflight results may be due to the fact that the spaceflight environment has far lower shear than LSMMG [17]. It may also be due to differences in EPS composition between *P. aeruginosa* PAO1 and PA14, where *P. aeruginosa* PA14 has Pel as a primary polysaccharide, *P. aeruginosa* PAO1 has Psl [50]. Because similar aggregation has been observed with *Salmonella* grown during spaceflight [13] and *S. aureus* grown in LSMMG [43], further studies of how shear, nutrient availability and genetics affect this behavior may provide additional insights into how different microbes respond to the spaceflight environment.

## Conclusions

Our findings indicate that differences in *P. aeruginosa* final cell densities observed between spaceflight and normal gravity are due to an interplay between changes in mixing and transport that occur under microgravity conditions and the availability of substrates essential for growth. These results may improve our ability to predict how different microbes will respond to microgravity and how combinations of environmental and genetic factors affect important behaviors, such as growth and virulence, both during spaceflight and on Earth. Here, we have shown that the microgravity environment encountered during spaceflight appears to allow cells to grow better with limited resources when compared to similar conditions on Earth. Considering the majority of bacteria, including those that are found on spacecrafts and space stations, live in nutrient-limited environments, this finding highlights the need for ongoing studies of how these effects may affect crew health during long-term spaceflight.

## Methods

### Bacterial strains and culture media

*Pseudomonas aeruginosa* PA14 [23] and its isogenic motility mutant, *ΔmotABCD*[40], were used for the current experiment. All strains were grown overnight at 37°C with shaking at 225 rpm in nutrient broth (NB) (Difco BD). To prepare inocula, cultures were washed and resuspended in phosphate buffered saline (PBS) to a final concentration of ~6 x 10^6^ CFU/mL. The modified artificial urine medium (mAUM) used in the present study is based on the medium previously described by Brooks *et al.,*[31]. To ensure reproducibility in media composition, RPMI 1640 amino acid solution (50x; Sigma, cat #: R7131) and L-glutamine solution (200 mM; Sigma, cat #: G7513) were substituted for peptone and yeast extract. The concentration of calcium chloride dihydrate was lowered from 2.5 mM to 0.25 mM to minimize precipitation during storage at 8°C. Sodium nitrate, 6 mM, was added to serve as a terminal electron acceptor and enable some growth under oxygen limiting conditions. Higher concentrations of sodium nitrate were avoided to minimize any effects on aerobic growth and limit the accumulation of nitrite, which can inhibit cell growth. To test the effects of carbon source, 2 mM citric acid was substituted by 2 mM glucose (mAUMg). To assess the role of phosphate availability, we used either 5 mM or 50 mM phosphate, where mAUM and mAUMg include 5 mM phosphate and mAUM-high Pi and mAUMg-high Pi contain 50 mM phosphate. The pH of all media was adjusted to 7.0.

### Spaceflight and ground control cultures

Spaceflight and ground control cultures of *P. aeruginosa* were prepared and grown in a fluid processing apparatus (FPA) as described previously [24]. To load the FPA, a 13-mm diameter mixed cellulose ester membrane disc (Millipore) was first attached to a solid or a gas exchange (GE) insert with autoclavable double-sided tape (3 M). Next, 2.5 mL of media and 0.5 mL of inoculum were sequentially loaded into two compartments separated by a rubber stopper. Due to constraints imposed by the dimensions of the bevel, approximately 0.6 mL of air remained in the compartment containing the media after loading. A third compartment, for FCM samples only, was filled with 2.4 mL of 9% w/v paraformaldehyde in PBS. Three biological replicates were prepared for each experimental condition. Eight FPAs were loaded into a single group activation pack (GAP), which enables simultaneous plunging of the rubber stoppers in each FPA, and were incubated in a Commercial Generic Bioprocessing Apparatus (CGBA) as described previously [30]. Cultures were grown following the experiment timeline shown in Figure 1. Before shuttle launch, all FPAs were loaded and stored at ambient temperature for at least 48 h to check for contamination. Samples were subsequently stored at 8°C (L-1) until activation. Five days before shuttle landing, the media and inoculum were combined and incubated at 37°C for 72 h (activation). Two days before shuttle landing, the temperature was decreased to 8°C and the FCM samples were fixed with paraformaldehyde (termination). Samples were obtained approximately 6 h after shuttle landing and processed immediately. Ground controls were conducted at Kennedy Space Center in parallel with spaceflight samples. The attached membranes were separated from the liquid phase, planktonic fractions. Characterization and analysis of biofilm growth on the membranes have been reported separately [24].

### Plate counting

One mL of the planktonic fraction was sonicated in an ultrasonic bath (Branson 2510) for 8 min to disperse any clumps of cells. The planktonic fraction was serially diluted with PBS. Samples were spot-plated on NB agar and incubated at 37°C for 18 h prior to counting.

### Sample preparation and flow cytometric analysis

Bacterial cells fixed with paraformaldehyde were washed with PBS and stained with 4′,6-diamidino-2-phenylindole (DAPI; Life Technologies) for 2 h at room temperature according to the manufacturer’s instructions. Stained samples were diluted to approximately 5 x 10^5^ – 9 x 10^6^ CFU/mL with PBS. Cell numbers were measured using a FACSAria flow cytometer (BD Biosciences) equipped with a 407 nm laser. To calculate the concentration of cells from FCM recorded events, liquid counting beads (BD Biosciences) were used following the manufacturer’s instruction. Briefly, 50 μL of the liquid counting beads was added to 1 mL of each diluted sample. The beads were detected by FCM in a separate region that does not overlap with the cells. The number of events in the cell region was recorded until one thousand events in the bead region were detected. The concentration of cells in each sample was calculated using the following equation.

$$\begin{array}{ll} \text{Conc}.\;\mathrm{of}\;\text{cells}= & \frac{\#\;\mathrm{of}\;\text{events}\;\mathrm{in}\;\text{cell}\;\text{region}}{\#\;\mathrm{of}\;\text{events}\;\mathrm{in}\;\text{bead}\;\text{region}}\\ & \;x\frac{\#\;\mathrm{of}\;\text{beads}/\;\text{test}}{\text{test}\;\text{volume}}x\;\text{dilution}\;\text{factor} \end{array}$$

### Effects of post-growth storage on viable cell counts and FCM data

The experiments were performed using FPAs following the same timeline and temperature profile as described in Figure 1. FPAs were incubated at 37°C for 72 h. Samples were either processed immediately or stored at 8°C for 48 h. To process samples, 1 mL of culture was enumerated by plate counting and a second 1 mL fraction was fixed with paraformaldehyde and enumerated by FCM.

### Light microscopy

For differential interference contrast (DIC) microscopy, *P. aeruginosa* stains fixed with paraformaldehyde after experimental termination. The fixed *P. aeruginosa* stains were observed using Olympus IX-81 inverted microscope equipped with DIC optics, 60x/1.35 oil Zeiss.

### Statistical analysis and numerical interpretation

All data were represented as mean ± standard deviation (S.D.) of three biological replicates. Statistical comparison of the number of cells between normal gravity and spaceflight for each different condition was conducted using a one-tailed Student’s t-test. When assessing combinations of two factors (post-growth storage and the microgravity environment, or phosphate concentration in media and microgravity) or three factors (motility, phosphate concentration in media, and microgravity), the effects of each factor and their interaction were assessed by two or three-way ANOVA with post-hoc Tukey tests using PASW Statistics18 (SPSS Inc.). Statistical significance was defined as *p* < 0.05.

## Competing interests

The authors declare that they have no competing interests.

## Authors’ contributions

WK, FKT, JSD, JLP and CHC designed the study and prepared this manuscript. WK, FKT, JS, NM, HKC, ZY, RP, MP, JLP and CHC performed the experiments. WK, FKT, ZY, HKC, and CHC analyzed the results. All authors read and approved the final manuscript.

## Supplementary Material

Additional file 1: Figure S1 Illustration of fluid processing apparatus (FPA). Click here for file
