# Supplementary material for: Effect of spaceflight on Pseudomonas aeruginosa final cell density is modulated by nutrient and oxygen availability
Source: BMC Microbiol. 2013 Nov 6;13:241. doi: 10.1186/1471-2180-13-241 (PMC4228280; doi:10.1186/1471-2180-13-241)
Supplement: Additional file 1: Figure S1 — Illustration of fluid processing apparatus (FPA). [file 1471-2180-13-241-S1.pdf]

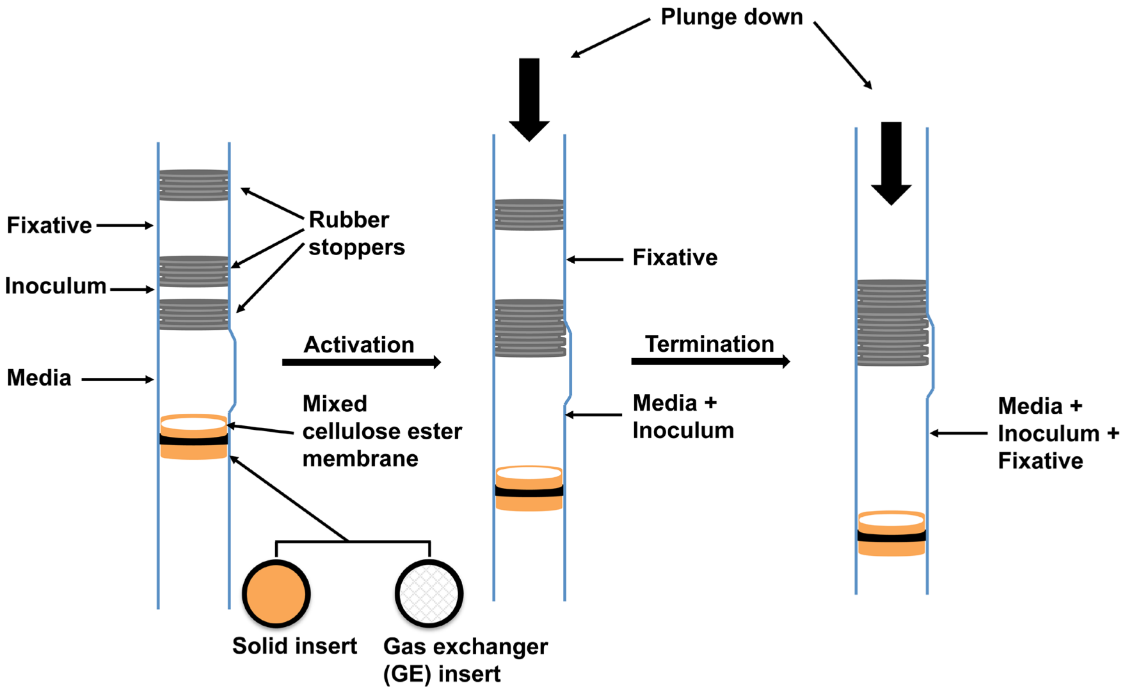

Supplementary Figure S1. Illustration of fluid processing apparatus (FPA). Each FPA consists of a glass barrel, rubber stoppers, and a solid insert or a gas exchange (GE) insert. A mixed cellulose ester membrane was attached with double-sided tape to either a solid or a GE insert. 2.5 mL of media and 0.5 mL of inoculum were subsequently loaded into FPAs, separated by rubber stoppers. For flow cytometry samples only, 2.4 mL of 9% (w/v) solution of paraformaldehyde in PBS was loaded into the third compartment. Compartments were mixed by downward plunging.
